# Does intervention with GLP-1 receptor agonist semaglutide modulate perception of sweet taste in women with obesity: study protocol of a randomized, single-blinded, placebo-controlled clinical trial

**DOI:** 10.1186/s13063-021-05442-y

**Published:** 2021-07-19

**Authors:** Mojca Jensterle, Simona Ferjan, Tadej Battelino, Jernej Kovač, Saba Battelino, Dušan Šuput, Andrej Vovk, Andrej Janež

**Affiliations:** 1 Department of Endocrinology, Diabetes and Metabolic Diseases, Division of Internal Medicine, University Medical Centre Ljubljana, Zaloška cesta 7, SI-1000 Ljubljana, Slovenia; 2 Faculty of Medicine, University of Ljubljana, Vrazov trg 2, SI-1000 Ljubljana, Slovenia; 3 Department of Endocrinology, Diabetes and Metabolism, University Children’s Hospital, University Medical Centre Ljubljana, Bohoričeva 20, SI-1000 Ljubljana, Slovenia; 4 Department of Otorhinolaryngology and Cervicofacial Surgery, University Medical Centre Ljubljana, Zaloška cesta 2, SI-1000 Ljubljana, Slovenia

**Keywords:** Semaglutide, Taste, Gustatory coding, PCOS, Obesity

## Abstract

**Background:**

Preclinical studies demonstrated that glucagon-like peptide 1 (GLP-1) is locally synthesized in taste bud cells and that GLP-1 receptor exists on the gustatory nerves in close proximity to GLP-1-containing taste bud cells. This local paracrine GLP-1 signalling seems to be specifically involved in the perception of sweets. However, the role of GLP-1 in taste perception remains largely unaddressed in clinical studies. Whether any weight-reducing effects of GLP-1 receptor agonists are mediated through the modulation of taste perception is currently unknown.

**Methods and analysis:**

This is an investigator-initiated, randomized single-blind, placebo-controlled clinical trial. We will enrol 30 women with obesity and polycystic ovary syndrome (PCOS). Participants will be randomized in a 1:1 ratio to either semaglutide 1.0 mg or placebo for 16 weeks. The primary endpoints are alteration of transcriptomic profile of tongue tissue as changes in expression level from baseline to follow-up after 16 weeks of treatment, measured by RNA sequencing, and change in taste sensitivity as detected by chemical gustometry. Secondary endpoints include change in neural response to visual food cues and to sweet-tasting substances as assessed by functional MRI, change in body weight, change in fat mass and change in eating behaviour and food intake.

**Discussion:**

This is the first study to investigate the role of semaglutide on taste perception, along with a neural response to visual food cues in reward processing regions. The study may identify the tongue and the taste perception as a novel target for GLP-1 receptor agonists.

**Ethics and disseminations:**

The study has been approved by the Slovene National Medical Ethics Committee and will be conducted in accordance with the Declaration of Helsinki and Good Clinical Practice guidelines. Results will be submitted for publication in an international peer-reviewed scientific journal.

**Trial registration:**

ClinicalTrials.govNCT04263415. Retrospectively registered on 10 February 2020

**Supplementary Information:**

The online version contains supplementary material available at 10.1186/s13063-021-05442-y.

## Background

“Feeling good” is one of the dominant drives in mammalian physiological systems [1], and feeding is one of the fundamental processes which induce this sensation. By eating, we not only ingest nutritive calories but also use those calories to stimulate a hedonic feeling. Usually, the most rewarding feelings are offered by calorically dense foods that taste sweet and fat [2]. In societies where such foods are readily available with little or no effort, many individuals cannot control their eating habits and consume food in excessive amounts [2].

The human taste is, after the visual and olfactory sensations, the first potential gateway for controlling qualitative and quantitative features of food intake [3, 4]. It conveys important information about the perceptual quality and about an appetitive and hedonic value of the ingested substances [3, 4]. It analyses chemosensory (modality, intensity), orosensory (texture, temperature, pungency) and rewarding properties of food [4].

Alterations in metabolic health can significantly affect taste perception [5, 6]. Obese individuals often perceive sweet tastes as less intense and may need more sweet-tasting agents to satisfy their reward producing need for sweet [7]. Reciprocally, populations that are prone to obesity have been shown to have an inherently elevated desire for sweet diets [8]. The mechanisms behind these alterations are not well elucidated.

Preclinical research provided some interesting insights into the direct role of an incretin glucagon-like peptide-1 (GLP-1) in the gustatory coding. It was found that (1) GLP-1 is locally synthesized in the subpopulations of taste bud cells (TBCs) in the tongue, (2) GLP-1 receptor (GLP-1 R) exists on the gustatory nerve fibres in close proximity to GLP-1-containing taste bud cells and (3) this paracrine GLP-1 signalling is specifically involved in the perception of sweet taste [2, 9–12]. Stimulation of the cognate receptors on the taste bud cells with sweet compound results in a release of GLP-1. Higher concentrations of sweet stimuli produced higher GLP-1 stimulation [12]. The released GLP-1 activated GLP-1 R on the gustatory nerve fibres in close proximity to GLP-1-containing TBCs. Functionally, GLP-1 acts as an important neurotransmitter in peripheral taste coding for the perceptual experience of the sweet [2, 9–12].

From the taste bud, gustatory nerve fibres further transmit the gustatory signal to the nucleus solitary tract (NTS) in the medulla. NTS is an important site of convergence for gustatory fibres. Its fibres synapse with thalamic neurons. Thalamic fibres project to the primary gustatory cortex in the insula, where taste qualities are further distinguished [3, 4]. Remarkably, NTS neurons are also the main source of locally produced GLP-1 in the brain. GLP-1 from NTS is widely distributed to GLP-1 R in the hypothalamus and mesolimbic system that are involved in the perception of the reward value of the food and in the regulation of food intake [2].

However, despite two very strategic locations on a trajectory of the gustatory coding—firstly, in the taste bud cells and, secondly, in the NTS—this specific role of locally produced GLP-1 remains largely unexplored.

Given an established role of GLP-1 receptor agonists (GLP-1 RAs) in reducing body weight in people who are overweight or obese, with or without diabetes [13–17], it is therefore reasonable to question whether any weight-reducing effects of GLP-1 RAs are mediated trough the modulation of gustatory coding.

Semaglutide is a human GLP-1 analogue that has 94% structural homology with native human GLP-1 and three important modifications [18] which extend its half-life in humans to approximately 1 week [18]. Treatment with semaglutide results in significant weight loss, reduced energy intake, decreased appetite and food carvings and decreased relative preference for energy-dense foods [19].

Based on this collective evidence, we aim to investigate whether semaglutide modulates gustatory coding along with the changes of food preference and consumption ensue.

The following are the objectives of the study:

- ➢ Objective 1: To investigate whether treatment with semaglutide in women with PCOS and obesity will modulate microenvironment of the taste buds as assessed by transcriptomic profile (RNA-seq)
- ➢ Objective 2: To investigate whether treatment with semaglutide will enhance the sensitivity for sweet taste
- ➢ Objective 3: To investigate whether treatment with semaglutide will modulate neural response to visual food cues and to sweet-tasting substances in reward processing regions
- ➢ Objective 4: To investigate whether treatment with semaglutide will alter food preference

## Methods and analysis

The SPIRIT reporting guidelines was used in the protocol [20].

### Study design

To test the hypothesis, we designed a 16-week, single-blinded, randomized, placebo-controlled clinical trial, to compare the effects of semaglutide versus placebo in women, diagnosed with obesity and polycystic ovarian syndrome (PCOS). The women will be randomized in a 1:1 ratio to the semaglutide or placebo group.

### Study setting

The patients with PCOS and obesity will be recruited from the academic outpatient clinics in University Medical Centre, Ljubljana, Slovenia.

### Study population and eligibility criteria

We reasoned to choose as patients’ cohort a group of healthy obese women with polycystic ovary syndrome (PCOS) that have increased metabolic risk, in order to avoid any other concomitant clinical feature as a confounding factor, and potentially affecting the results of our study. The diagnosis of PCOS will be established using the Rotterdam criteria, specifically phenotype A, characterized as concomitant presence of irregular menstrual cycles, hyperandrogenism, and polycystic ovarian morphology [21]. We require phenotype A to increase the homogeneity of the included population. Irregular menstrual cycles are defined as: < 21- or > 35-day-long cycle or < 8 cycles per year or > 90 days length for any one cycle. Clinical hyperandrogenism includes hirsutism assessed by modified Ferriman Gallway score with a level ≥ 6, acknowledging that self-treatment is common and can limit clinical assessment; alopecia preferably assessed by Ludwig visual score; and acne without universally accepted visual assessments for evaluating acne. Assessment of biochemical hyperandrogenism will include calculated free testosterone, free androgen index or calculated bioavailable testosterone. Androstenedione and dehydroepiandrosterone sulphate (DHEAS) would be considered if testosterone would not be elevated. Assessment of biochemical hyperandrogenism would be useful in establishing the diagnosis of PCOS and/or phenotype where clinical signs of hyperandrogenism (in particular hirsutism) would not be unclear or absent. Using endovaginal ultrasound transducers with a frequency bandwidth that includes 8 MHz, the threshold for polycystic ovarian morphology (PCOM) on either ovary would be a follicle number per ovary of 20 and/or an ovarian volume ≥ 10 ml on either ovary, ensuring no corpora lutea, cysts or dominant follicles are present. If using older technology, the threshold for PCOM could be an ovarian volume ≥ 10 ml on either ovary. Obesity is defined as BMI ≥ 30 kg/m^2^. We will enrol expectedly 30 participants. Eligible patients are adults (age 18 to menopause), with obesity (BMI ≥ 30 kg/m^2^) and no known serious chronic illness (including type 1 or 2 diabetes). Inclusion and exclusion criteria are listed in Table 1.

**Table 1:** Eligibility criteria for participants

**Inclusion criteria** ► PCOS as diagnosed by the Rotterdam criteria ► BMI ≥ 30 kg/m^2^ ► Age 18–45 years ► Informed written consent	
**Exclusion criteria** ► Patients diagnosed with any known serious chronic illness, including type 1 or 2 diabetes (or a randomly measured fasting plasma glucose > 7 mmol/l) ► Angina pectoris, coronary heart disease or congestive heart failure (NYHA Classes III–IV) ► Severe renal impairment (creatinine clearance (GFR) < 30 mL/min) ► Severe hepatic impairment ► Inflammatory bowel disease ► Gastroparesis ► Cancer ► Chronic obstructive lung disease ► Psychiatric disease, a history of major depressive or other severe psychiatric disorders ►Bleeding diathesis or anticoagulant treatment ► Current or history of neurological disease including traumatic brain injury ► Contraindications for MR scanning (implants, claustrophobia, etc.) ► The use of medications that cause clinically significant weight gain or loss ► Previous bariatric surgery ► A history of idiopathic acute pancreatitis ► A family or personal history of multiple endocrine neoplasia type 2 or familial medullary thyroid carcinoma ► Current smoking ► Pregnancy, expecting pregnancy or breastfeeding ► Allergy to any of the ingredients of the study medication: semaglutide, disodium phosphate dihydrate, propylene glycol, phenol, hydrochloric acid and sodium hydroxide ► Anticipated change in lifestyle (e.g. eating, exercise or sleeping pattern) during the trial ► Any condition that the investigator feels would interfere with trial participation (such as inability to follow frequent trial visits according to protocol schedule)	

*Abbreviations*: *PCOS* polycystic ovary syndrome, *BMI* body mass index, *GFR* glomerular filtration rate, *MR scanning* magnetic resonance scanning

### Recruitment strategy

We will identify patients diagnosed with PCOS referred to the first endocrine check-up. Since we are a national referral centre for patients with PCOS, the recruiting pool is expected to be sufficient. The recruitment procedure starts as a pre-screening when the patients from the hospital register are contacted by the investigator. All potentially eligible patients will receive written and oral information about the study. Screening examination will be performed after the patient has agreed to participate and has signed the informed consent form. At the time of screening, the patients will undergo examinations to assure that all inclusion criteria and none of the exclusion criteria are met (Table 1). All patients who meet the inclusion and exclusion criteria at screening will be enrolled for randomization followed by a 16-week treatment period. The inclusion frequency will be regularly evaluated.

### Withdrawal criteria

Patients are free to withdraw from the study at any time without providing a reason therefore and without any impact on further treatment at the UMC Ljubljana outpatient clinics. The reason for dropout maybe withdrawal of consent, adverse event, pregnancy discovered during the trial or failure to comply with clinical trial medication or protocol. If the patient misses more than one injection or more than one visit in total, she will be excluded.

### Randomization

The patients will be randomized into two groups using the RAND randomization function. The random allocation sequence will be generated by an extern statistician.

### Blinding

The study personnel responsible for the group randomization and the main investigator will not be blinded. Other participating personnel, including patients, outcome assessors and persons performing the data analysis, will remain blinded from the time of randomization to the time of database unlock.

### Unblinding

Unblinding of the participants will be permissible under the following circumstances: treatment of a participant in a medical emergency that requires knowledge of treatment allocation, treatment of a participant for an adverse event (AE), in the event of a suspected unexpected serious adverse reaction (SAR), and SAR is defined as any untoward medical occurrence that results in death, is life-threatening, requires hospitalization or prolongation of existing hospitalization, results in persistent or significant disability/incapacity or is a congenital anomaly or birth defect.

### Implementation

This is a novel study with potential implementation for weight management and obesity-related disorders. Investigating the changes in food and taste perception is of paramount interest to further understand the mechanisms of drug treatments for obesity.

### Patient and public involvement

No patients were involved in the development of the research question or in designing the study, and the burden of the intervention is not assessed by patients themselves. Patients will receive the clinical biochemical and imaging results of the study. We will discuss the test results with the participants in plain language after their endpoint assessments are completed.

### Sample size calculation

Setting the targeted statistical power of analysis at 90% with two-sided test alpha (*α*) value of 0.05 and aiming to gain at least 10 reads per gene for > 90% of all annotated genes and assuming the level of biological variation at 0.5, the required number of samples per group to detect a 2-fold change in gene expression is 15, following the recommendations reported by Hart and colleagues [22].

### Interventions

The GLP-1 RA semaglutide (0.5 mg followed by 1 mg) (Ozempyc, Novo Nordisk A/S Bagsvaerd, Denmark) or placebo will be administered once weekly as subcutaneous injections in the abdomen or thigh. Subjects will receive either semaglutide (1.34 mg/ml) or a matching placebo. The placebo will be supplied as prefilled saline syringes in the placebo pen and will be administrated in the same way and volume as semaglutide. The starting dose will be 0.5 mg (4 weeks) escalating to 1.0 mg (12 weeks) administrated subcutaneously (s.c.) once weekly. The starting dose will be 0.5 mg (4 weeks) escalating to 1.0 mg administrated subcutaneously (s.c.) once weekly for 12 weeks. A 16-week time point is the earliest time frame to assess the effect of GLP-1 RAs on body mass in those who respond to therapy. We hypothesize that the expected changes in body mass weight also reflects in taste perception and neural response. The injection should be given before bedtime, before 10 p.m. Subjects will attend the clinics for the dose escalation. They will be reminded by text message to administer the drug at home.

### Treatment allocation

After the screening phase, participants will be randomized after the test day to one of the two study groups in a 1:1 ratio in accordance with a subject randomization list (SRL). A non-blinded study nurse (not otherwise associated with the trial) will allocate study participants according to the SRL. Each box of study medication will be labelled with a unique number (UN).

### Trial visits and examinations

At screening (visit 0), a general assessment of inclusion and exclusion criteria, demography, medical history and concomitant medications will be recorded. Patients will receive oral and written information about the study. At visit 1 (randomization), the enrolled patients will undergo physical examination including antropometric measurements (weight, height and waist circumference) and blood pressure measurement, together with blood samples for standard endocrine and biochemical measurements. Assessment of whole-body composition will be measured by hologic dual-energy X-ray absorptiometry (DXA), including the visceral adipose tissue (VAT) area. A taste test and tongue tissue biopsy will be performed. Eating behaviour and foot preference will be assessed by the Three-Factor Eating Questionnaire (TFEQ-R18) and Slovenian Food Preference Questionnaire, respectively. To assess food intake, a 3-day diet diary will be completed. After visit 1, the gastric emptying test and functional MRI will be performed within 1 week (visit 2). Identical test as in visits 1 and 2 will be made at the end of intervention (visits 6 and 7). According to randomization at the 3rd visit, participants will start with semaglutide or placebo therapy. At visit 4, clinical control with adverse effect record will be made, and at visit 5, dose escalation will be made.

**Fig. 1:**
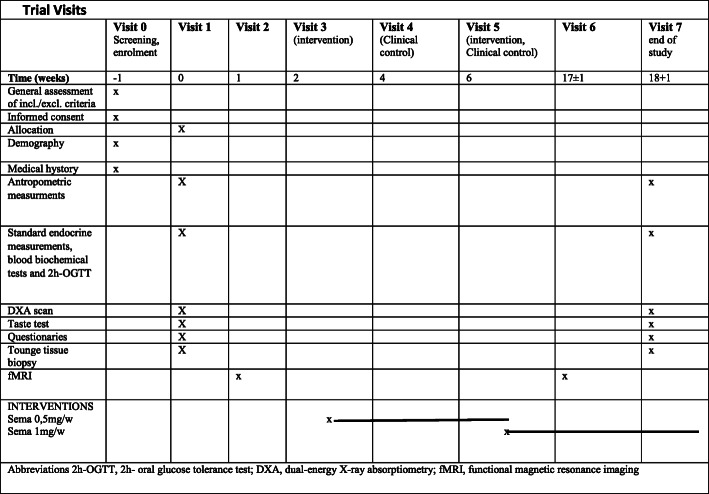
Trial visits. Abbreviations: incl./excl. criteria, including/excluding criteria; 2h-OGTT, 2-h oral glucose tolerance test; DXA, dual-energy X-ray absorptiometry; fMRI, functional magnetic resonance imaging; Sema 0.25mg/w, semaglutide 0.25 mg per week; Sema 0.5mg/w, semaglutide 0.5 mg per week; Sema 1mg/w, semaglutide 1 mg per week

The list of potential side effects is provided in Table 2. The patients will be advised to contact the study team if they encounter any problems or adverse effects between the visits V3–7. The time schedule of enrolment, visits and assessments is presented in a schematic diagram (Fig. 1).

**Table 2:** The list of potential side effects

**Nervous system disorders** ► Dizziness ► Dysgeusia **Cardiac disorders** ► Increased heart rate **Gastrointestinal disorders** ► Nausea ► Diarrhoea ► Vomiting ► Abdominal pain ► Abdominal distension ► Constipation ► Dyspepsia ► Gastritis ► Gastro-oesophagealreflux diseaseafe ► Eructation ► Flatulence ► Acute pancreatitis **Hepatobiliary disorders** ► Cholelithiasis **General disorders and administration site conditions** ► Fatigue ► Injection site reactions

### Auditing

External auditing is not planned.

### Outcomes

The objective of the present protocol is to evaluate the effect of semaglutide on gustatory coding.

#### Primary endpoint

The primary endpoints are alteration of transcriptomic profile of human tongue tissue as changes in expression level from baseline to follow up after 16 weeks of treatment, measured by RNA sequencing, and change in taste sensitivity as detected by chemical gustometry.

#### Secondary endpoints

Secondary endpoints include change in neural response to visual food cues in reward processing regions as assessed by functional MRI, change in body weight, change in fat mass as determined by DXA scan and change in eating behaviour.

The expected change for bodyweight reduction is between 6 and 8%. The expected change for taste perception as assessed by chemical gustometry is about 25% within the category for a specific taste. We have no quantitative predictions for the other endpoints.

Primary and secondary endpoints are shown in Table 3.

**Table 3:** Outcomes

**Primary endpoint** ► Alteration of transcriptomic profile of human tongue tissue as changes in expression level from baseline to follow-up after 16 weeks of treatment, measured by RNA sequencing ►Change in taste sensitivity as detected by chemical gustometry	
**Secondary endpoints** ► Change in neural response to visual food cues in reward processing regions as assessed by functional MRI ► Change in body weight ► Change in fat mass as determined by DXA ► Change in eating behaviour	

*Abbreviations*: *RNA sequencing* ribonucleic acid sequencing, *DXA* dual-energy X-ray absorptiometry, *functional MRI* functional magnetic resonance imaging

### Assessments

#### Menstrual frequencies

Menstrual regularity was defined as the number of bleeds per year using self-reported menstrual intervals based on diary review.

#### Anthropometrics

Height will be measured with a wall-mounted stadiometer to the nearest ± 1 cm. Bodyweight will be measured with a body weight scale to the nearest 1 kg. Body mass index will be calculated as the weight in kilogrammes divided by the square of height in metres. Waist circumference will be measured with 250-cm-long non-elastic measurement tape.

#### DXA scan

The whole-body composition will be assessed by a dual-energy X-ray absorptiometry (Discovery A; Hologic, Waltham, MA) with the software provided by the manufacturer (QDR for Windows version 12.5). The instrument generates values for whole-body fat mass, lean body mass and bone mineral content and all these parameters separately for different body parts.

#### Blood pressure

Blood pressure and resting heart rate will be measured in duplicate from the non-dominant arm with a digital blood pressure monitor (Omron M3, Intellisense (HEM-75051-E), Omron Healthcare Europe B.V., The Netherlands) in a sitting position after at least 5 min of rest.

#### Blood analysis

Participants must be fasting for a minimum of 10 h prior to test days, including foods, liquids and medications (except study medication).

Glucose levels will be determined using a standard glucose oxidase method (Beckman Coulter Glucose Analyzer, Beckman Coulter Inc., CA, USA). Androstenedione and DHEAS will be measured by specific double antibody RIA using 125 I-labelled hormones (Diagnostic Systems Laboratories, Webster, TX). Total and free testosterone levels will be measured by coated tube RIA (DiaSorin, S. p. A, Salluggia, Italy, and Diagnostic Products Corporation, LA, respectively). LH and FSH will be measured using an immunometric assay (Diagnostic Products Corporation, LA). Intra-assay coefficient variation (CV) for androstenedione ranges from 5.0 to7.5% and inter-assay CV from 4.1 to 11.3%, intra-assay CV for DHEAS from 4.9 to 9.8% and inter-assay CV from 7.9 to 13.0%. Intra-assay CV for free testosterone is 7.7–19.3%, and inter-assay CV is 6.4–13.2%. Intra-assay CV for total testosterone is 5.1–16.3%, and inter-assay CV is 7.2–24.3%. Intra-assay CV for SHBG is 2.5–5.3%, and inter-assay CV is 4–6.6%. BMI will be calculated as the weight in kilogrammes divided by the square of height in metres. Menstrual regularity will be defined as the number of bleeds per year using self-reported menstrual intervals based on diary review. According to the World Health Organization (WHO) criteria, available from http://www.who.int/diabetes/publications/diagnosis_diabetes2006/en/, normal glucose tolerance (NGT) will be defined as fasting glucose levels below 6.1 mmol/l and 2-h glucose in 75 g-OGTT < 7.8 mmol/l, impaired fasting glucose (IFG) as fasting glucose between 6.1 and 6.9 mmol/l and 2-h glucose in 75 g-OGTT < 7.8 mmol/l, impaired glucose tolerance (IGT) will be identified by 2 h glucose levels in 75 g-OGTT between 7.8 and 11 mmol/l and T2D as fasting glucose level ≥ 7.0 mmol/l or 2-h glucose ≥ 11.1 mmol/l during OGTT.

### Questionnaires to assess eating behaviour, food preference and food intake

#### Eating behaviour

Eating behaviour will be assessed by using a Slovenian translation of the Three-Factor Eating Questionnaire (TFEQ-R18) at visit 1 and visit 8 [23]. The instrument is a shortened and revised version of the original 51-item TFEQ [24]. The translation of the Slovenian version had been back-translated by a native English speaker and evaluated as required. The questionnaire measures three different aspects of eating behaviour: cognitive restraint (CR) referring to the conscious restriction of food intake in order to control body weight or to promote weight loss, uncontrolled eating (UE) referring to the tendency to eat more than usual due to a loss of control over intake accompanied by subjective feelings of hunger and emotional eating (EE) referring to the inability to resist emotional cues. The TFEQ-R18 consists of 18 items on a 4-point response scale (definitely true/mostly true/mostly false/definitely false). Responses to each of the 18 items are given a score between 1 and 4, and item scores are summed up into scale scores for CR, UE and EE [23, 24]. The raw scale scores are transformed to a 0–100 scale [raw score_lowest possible raw score/possible raw score range_100], and the commonly used “half-scale” method is utilized to compensate for missing data on some items. Higher scores in the respective scales are indicative of greater CR, UE or EE.

#### Food preferences

Food preferences will be measured in a fasted state with the Slovenian Food Preference Questionnaire at visit 1 and visit 8. This is a task where standardized pictures of 68 typical Slovenian food items are shown in the following categories: high-fat savoury, low-fat savoury, high-fat sweet and low-fat sweet. Participants are instructed to rate each individual food item according to liking and to systematically choose the food items they prefer the most.

#### Food intake

To assess normal eating habits, instructions will be provided to record all food and beverages consumed on 2 working days and 1 weekend day in a 3-day food diary. The types and amount of food consumed will be recorded by participants in household measures (e.g. teaspoons, tablespoons) or using the weight listed on food and beverage packaging. Data will be entered to the Nutritics Professional Nutrition Analysis Software, version 4.267, Research Edition (Nutritics, Dublin, Ireland, www.nutritics.com). Participant’s mean daily nutrient intakes will be calculated in macronutrients as percentages of total energy intake for pre- and post-intervention. Food diaries will be administrated at visit 1 and visit 8.

### Chemical gustometry

Taste sensitivity will be evaluated with the “taste strips” test (https://www.burghart-mt.de/en/) which is a validated examination method to determine the perception of four basic tastes. The test consists of 16 taste strips consisting of 4 different concentrations of sweet, 4 different concentrations of sour, 4 different concentrations of salty and 4 different concentrations of bitter substances. The strips will be placed on the patient’s tongue in a pseudo-randomized manner, in accordance with the manufacturer’s instructions. The patient will be asked to identify the taste of each taste strip. The possible answer will be “sweet”, “sour”, “salty”, “biter” and “no taste”. The answer will be noted in the summary paper. Evaluation will be made with the manufacturer’s evaluation table.

### Neuroimaging data acquisition

Neuroimaging data will be acquired with a Achieva 3.0T TX scanner (Philips Healthcare, Best, The Netherlands). MR imaging is divided into 4 sessions; two were performed in the 2nd visit and two in the 6th visit after the treatment.

**Fig. 2:**
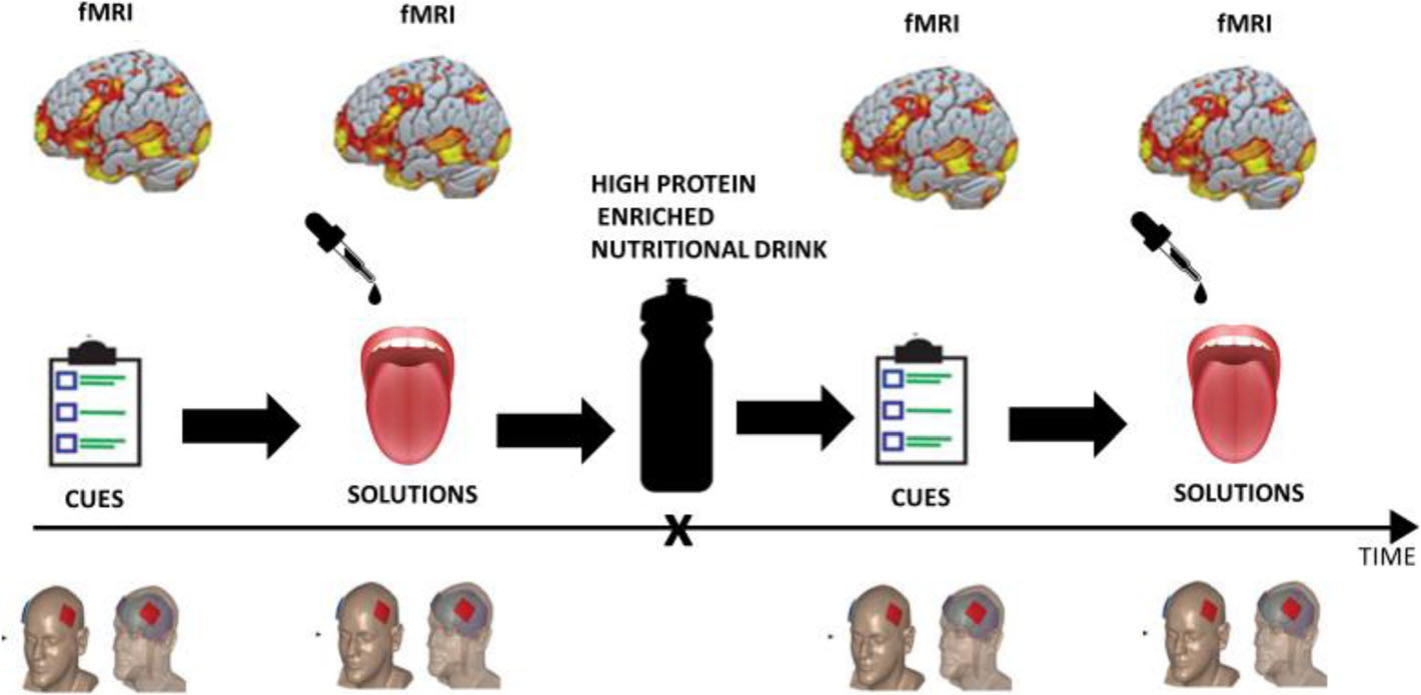
The procedure for neuroimaging data acquisitions

The patients in the fasting state will undergo two different tasks presented in block paradigms to maximize sensitivity for blood oxygen level-dependent signal change. In the first task, the patients will be shown a series of calorie-dense food cues, calorie-low food cues and non-food cues to assess inhibitory control. In the second task, the neural responses to three different solutions will be observed. The sweet solution will be prepared with dissolving glucose in distilled water, and the umami taste will be triggered with MSG dissolved in distilled water. The third, control solution that will also serve to rinse the taste, will be just distilled water. (82.5 g of glucose 0.83 M dissolved in 500 ml of distilled water; 8.45 g of MSG 0.1 M dissolved in 500 ml of distilled water). Both tasks will be repeated 30 min after ingested Prosure high-protein-enriched nutritional drink (Fig. 2). The fMRI will be performed in the fasting state and repeated after Prosure high-protein-enriched nutritional drink because post-ingestive effects can alter taste responses throughout the brain. When a particular food is eaten to satiety, it becomes less rewarding, without changing the taste of the food itself. In other words, the relative reward value of that particular food might diminish while the reward value of other foods can remain unchanged. Sensory-specific satiety changes have been observed both in electrophysiological studies on non-human primates and in human fMRI studies [25].

### Tongue tissue biopsy

Biopsies of the tongue will be taken as previously described by Archer and collogues [26] under sterile conditions and local anaesthesia with 2% lidocaine. Briefly, the tongue tissue will be cut from the anterior tongue (1 cm from the tongue apex and to the left). The procedure is well established and will be performed by a highly skilled ear, nose and throat (ENT) specialist. All samples will be put in a container with a mixture of water and formaldehyde and stored at − 80 °C until further processing.

### Tongue sample preparation and sequencing

Tongue biopsies stored in QIAzol lysis reagent (QIAGEN, Redwood 185 City, CA, USA) at − 80 °C will be thawed, resuspended and transferred to QIAshredder column (QIAGEN, Redwood 185 City, CA, USA) and homogenized by spinning at a maximum speed of microcentrifuge for 2 min. Homogenized lysate will be transferred to a Direct-zol RNA MiniPrep Plus spin column (Zymo Research, Irvine, USA), and RNA will be isolated by following the Direct-zol RNA Miniprep Plus manufacturer’s instructions. The purity of isolated RNA will be assessed using the DeNovix DS-11 FX+ spectrophotometer (DeNovix Inc., Wilmington, USA), and its integrity will be determined on an Agilent 2100 Bioanalyzer using the RNA 6000 Nano LabChip kit (Agilent, Santa Clara, USA). The RNA-seq library preparation will be performed by MGIEasy RNA Directional Library Prep Set (MGI Tech Co., Shenzen, China) with prior removal of ribosomal RNA by MGIEasy rRNA Depletion Kit (MGI Tech Co., Shenzen, China) following the manufacturer’s instructions. Data collection will run on a DNBSEQ-G400RS sequencer with DNBSEQ-G400RS High-throughput Sequencing Set (FCL PE150) (both MGI Tech Co., Shenzen, China). About 60 million of PE 150 reads will be collected per sample. Data and quality analysis will be performed by bcbio, python-based toolkit for genomic data analysis (https://github.com/bcbio/bcbio-nextgen). The reads will be aligned to reference by STAR aligner [27], and transcripts will be quantified by the salmon algorithm [28]. We will perform a panel analysis of 27 genes including ADIPOQ, AGRP, BDNF, EFNB1, GCG, GPL1R, LEP, LEPR, MC3R, MC4R, NPY, NPY1R, NPY5R, NR0B2, NTRK2, PCSK1, PMCH, POMC, PPARG, PYY, SIM1, SIRT1, SIRT3, TAS1R1, TAS1R2, TAS1R3 and UCP3.

### Ancillary and post-trial care

Post-trial care of the participants will include treatment with metformin in line with the latest international guidelines that advise a consideration of metformin as an adjunct to lifestyle intervention in adult women with PCOS with body mass index (BMI) ≥ 25 kg/m^2^, regardless of the presence of glucose disturbances and menstrual irregularity, for the management of weight and preventing or slowing progression to adverse cardiometabolic outcomes [21].

### Data collection, management and analysis

#### Data collection

Participants will be identified by study ID. Study data are collected and managed using the paper case report forms. Laboratory data will be transferred electronically from the laboratory performing clinical analyses and will be archived in secured hard drives with backup. All biological material (blood, tongue tissue) obtained from the study participants will be kept in a research bio-bank. Samples will be labelled with study ID. The bio-bank allows for the analyses of samples to be performed simultaneously to avoid large instrumental variations. The trial adheres to the Data Protection Act which requires data to be anonymized as soon as it is practical to do so.

#### Data analysis plan

Analyses will be based on two defined analysis sets: an intention-to-treat (ITT) analysis set and a per-protocol (PP) analysis set. ITT includes all randomized participants exposed to at least one dose of trial product that have completed the visit at week 2. The per-protocol population will include all patients completing the study with a documented valid baseline and end of treatment assessment of the primary endpoint without a major protocol violation. Safety analysis will be performed on the ITT analysis set. Two-tailed tests will be performed and the significance level will be set to *α* = 0.05.

#### Data management

All information concerning the subjects will be recorded and saved on password-protected computers. A detailed database will be set up to track each subject’s progress through this trial, including the scheduling of baseline and endpoint assessments. All the generated data will be stored on password-protected computers or servers that are only accessible to the research team.

#### Data statement

Data and resources will be shared with other eligible investigators through academically established means. The protocol and datasets used and/or analysed in this study will be available from the corresponding author on reasonable request. Researchers who provide a methodologically sound proposal may access data to achieve aims in the approved proposal.

#### Statistical analysis

The non-parametric Mann-Whitney test or Kruskal-Wallis test was used to compare the distribution of continuous variables between the different groups. The Wilcoxon signed-rank test was used for the comparison of continuous variables for related samples. Spearman’s rho (*ρ*) was used to assess the correlation between continuous variables. *P* values below 0.05 were considered statistically significant. Statistical analysis was performed using IBM SPSS Statistics, version 27.0 (IBM Corporation, Armonk, NY, USA).

The stability of differential gene expression will be controlled by cross-validation analysis of RNA-seq data. Differential expression (DE) between the groups will be analysed by application of edgeR and DESeq2 tools [29, 30]. Positive results will be controlled for false discovery rate (FDR) by the adaptive method of Benjamini et al. [31]. A list of differentially expressed genes with at least 2-fold difference in expression, called by both DE tools and passing the FDR test, will be collected, and Gene Ontology enrichment analysis will be performed to identify the potential signalling pathways and cellular processes of significance [32].

## Discussion

Taste perception has an impact on nutrient intake and termination of food consumption. The current evidence suggests that obesity alters gustatory coding, in particular, for sweet-tasting substances [7, 8, 33]. Weight reduction seems to increase taste acuity, yet the findings are inconsistent [33]. However, the role of taste perception in the development and persistence of obesity is currently unknown.

Data from preclinical studies suggest that locally produced GLP-1 in the taste bud cells acts as an ancillary but functionally important neurotransmitter in the initial coding of taste information and that it is specifically involved in the perception of sweet [2, 9–12]. Few interventional animal studies have shown that GLP-1 RA liraglutide decreased intake of foods high in sugar and fat [34, 35] and shifted food preferences towards an increase in the consumption of a chow diet and a decrease in candy intake [36]. Furthermore, GLP-1 RA exenatide decreased preference for sweet solutions but had no effect on other basic tastes [37].

The role of GLP-1 stimulation in human gustatory coding remains largely unexplored. It was reported that liraglutide modulated taste preference as assessed by visual analogue scale [38] and resulted in reduced desire to eat something sweet, salty, savoury or fatty [38]. Another study reported that liraglutide led to a decrease in the detection threshold for sweet tastes and decrease pleasure for fat food in overweight or obese poorly controlled individuals with type 2 diabetes. Liraglutide also induced a decrease in the detection threshold for bitter tastes. Collectively, liraglutide led to decreased carving for fatty foods and an enhanced sensitivity to sweet and bitter tastes without affecting the pleasure in eating [39]. In overweight/obese participants with type 1 diabetes, liraglutide modified macronutrient preferences with the decreased per cent of fat and surprisingly increased per cent of carbohydrates [40]. Recently, it was further demonstrated that semaglutide in individuals without diabetes lowers relative preference for fatty, energy-dense foods [19].

A series of studies reported that GLP-1 stimulation had modulated central nervous system response to visual and gustatory food cues as assessed by functional MRI [41–44]. It was demonstrated that endogenous GLP-1 and administration of GLP-1 RA affect CNS activation in response to viewing pictures of food items [42, 43], which is related to the predictive value of food consumption and craving for food. Responsiveness to the actual palatable food consumption demonstrated that GLP-1 induced increased central nervous system response to the ingestion of palatable foods [41, 44]. In individuals with type 2 diabetes, liraglutide decreased CNS activation after short- but not long-term intervention, suggesting that these effects of GLP-1 RA on the CNS may contribute to the induction of weight loss [44]. Another study indicated that emotional eaters have altered brain responses to food cues and are less sensitive to the central effects of GLP-1 receptor activation with an acute infusion of exenatide [45].

Until today, no interventional study with GLP-RAs addressed the potential modulation of the taste bud microenvironment. Due to the quick turnover and replacement of taste buds cell within 7–24 days [46, 47], we expect that the taste buds and the differentiating maturing taste cells would be highly responsive to the semaglutide-induced altered gene expression. If the taste perception demonstrates significant differences related to the GLP-1 RA therapy, we would like to further elucidate some mechanisms of this phenomenon. RNA expression analysis of the taste receptor genes and pathways could generate some data helping us in this endeavour. We expect that an altered tongue microenvironment will result in a change of taste sensitivity as detected by chemical gustometry along with a changed neural response to visual food cues in reward processing regions as assessed by functional MRI.

The main limitation of this study is that the analysed tongue tissue will be a collection of different cell types, including taste cells, epithelial cells, connective tissue and blood/immune cells. The potential differences in the gene expression will reflect the differences in the gene expression over some, or, all of these different cell types.

In conclusion, this is the first study to investigate the role of semaglutide on taste perception, along with a neural response to visual food cues in reward processing regions. The research may identify the tongue and the taste perception as novel treatment targets of GLP-1 RAs.

This field should initiate the collaboration of endocrinologists, diabteologists, ENT specialists, neuroscientists and nutritionists to further explore the role of GLP-1 in taste perception. Since consumption of calorie-dense palatable foods is highly pertinent to the onset and maintenance of obesity and diabetes, the potential modulation of taste sensitivity and food preference upon treatment with GLP-1-based therapies is of important clinical relevance. Exploring the potential possibilities to modulate gustatory coding by pharmacological manipulation remains one of an intriguing clinical challenges [48].

## Protocol amendments

This is protocol version 3 dated May 2020. We made further amendments on the basis of version 1 dated November 2019, and we will communicate the protocol amendments to the *Clinical Trials*. Slovenian Medical Ethics Committee has already been informed. Because of the COVID-19 pandemic, recruitment will delay to the end of May 2021 and is anticipated that the trial will be completed in October 2021.

## Supplementary Information

**Additional file 1.** Informed consent form for participation in the study.

**Additional file 2.** SPIRIT guidelines.

## Data Availability

The datasets supporting the findings of our study are available from the corresponding author upon reasonable request.

## Abbreviations

GLP-1: Glucagon-like peptide 1
PCOS: Polycystic ovary syndrome
RNA-seq: Ribonucleic acid sequencing
functional MRI: Functional magnetic resonance imaging
GLP-1 RAs: Glucagon-like peptide 1 receptor agonists
NTS: Nucleus solitary tract
DHEAS: Dehydroepiandrosterone sulphate
PCOM: Polycystic ovarian
BMI: Body mass index
AE: Adverse event
SAR: Serious adverse reaction
SRL: Subject randomization list
UN: Unique number
DXA: Dual-energy X-ray absorptiometry
VAT: Visceral adipose tissue
TFEQ-R18: Three-Factor Eating Questionnaire-R18
RIA: Radioimmunoassay
CV: Coefficient variation
NGT: Normal glucose tolerance
2h-OGTT: 2-h oral glucose tolerance test
IFG: Impaired fasting glucose
CR: Cognitive restraint
UE: Uncontrolled eating
EE: Emotional eating
MSG: Monosodium glutamate
ITT: Intention-to-treat
PP: Per-protocol
DE: Differential expression
FDR: False discovery rate
CNS: Central nervous system
