# Supplementary material for: Does intervention with GLP-1 receptor agonist semaglutide modulate perception of sweet taste in women with obesity: study protocol of a randomized, single-blinded, placebo-controlled clinical trial
Source: Trials. 2021 Jul 19;22:464. doi: 10.1186/s13063-021-05442-y (PMC8287101; doi:10.1186/s13063-021-05442-y)
Supplement: Supplementary file 1 — Additional file 1. Informed consent form for participation in the study. [file 13063_2021_5442_MOESM1_ESM.docx]

# Supplementary file 1

# Informed consent form for participation in the study

I have received oral and read written information regarding the study entitled “The effects of semaglutide on taste modulation, gastric emptying and the transmission of metabolotropic signals to the central nervous system in patients with polycystic ovarian syndrome (PCOS) and obesity: a randomized, single-blind, placebo-controlled clinical trial”. I have had the opportunity to discuss this study with the researchers and got any additional information that I requested.

I understand the purpose of the study and I am aware of the risks and disadvantages that may arise during the study as well as the benefits of the study. I will be informed of the possible abnormal results that may arise during the study.

I consent to participate in the study and I am aware that my participation is completely voluntary.

I agree with the access of researchers to the data in my medical records, and understand that the obtained data will be treated confidentially. I allow the use of anonymized data for research purposes.

I know that I can withdraw from the study at any time without affecting my health care in the future. I can ask for any remains of my biological samples to be destroyed and for all the corresponding data to be removed from the study database.

I understand that I will receive a copy of this information and the consent document

___________________________________________ ________________________
(Patient Signature) (Researcher Signature)

Name: ___________________________ ________________________
 (please print) (date)
